# Toxocariasis in North America: A Systematic Review

**DOI:** 10.1371/journal.pntd.0003116

**Published:** 2014-08-28

**Authors:** Rachel M. Lee, Laura B. Moore, Maria Elena Bottazzi, Peter J. Hotez

**Affiliations:** 1 Baylor College of Medicine, Houston, Texas, United States of America; 2 James A. Baker Institute of Public Policy, Rice University, Houston, Texas, United States of America; 3 Departments of Medicine, Pediatrics, and Molecular Virology and Microbiology, National School of Tropical Medicine, Baylor College of Medicine, Houston, Texas, United States of America; 4 Sabin Vaccine Institute and Texas Children's Hospital Center for Vaccine Development, Houston, Texas, United States of America; University of Melbourne, Australia

## Abstract

Toxocariasis is an important neglected tropical disease that can manifest as visceral or ocular larva migrans, or covert toxocariasis. All three forms pose a public health problem and cause significant morbidity in areas of high prevalence. To determine the burden of toxocariasis in North America, we conducted a systematic review of the literature following PRISMA guidelines. We found 18 articles with original prevalence, incidence, or case data for toxocariasis. Prevalence estimates ranged from 0.6% in a Canadian Inuit community to 30.8% in Mexican children with asthma. Commonly cited risk factors included: African-American race, poverty, male sex, and pet ownership or environmental contamination by animal feces. Increased prevalence of *Toxocara spp.* infection was linked in a group of case control studies conducted in Mexico to several high risk groups including waste pickers, asthmatic children, and inpatient psychiatry patients. Further research is needed to determine the true current burden of toxocariasis in North America; however the prevalence estimates gathered in this review suggest that the burden of disease is significant.

## Author Summary

Toxocariasis is a parasitic worm infection that is transmitted by the ingestion of parasite eggs present in the feces of dogs and cats. After ingestion of the Toxocara eggs, the released larvae migrate through human organs, including the lungs, liver, brain, and eyes, where they cause inflammation and organ damage. The different larval migrans syndromes associated with toxocariasis are diagnosed by detecting Toxocara antibodies in sera of infected individuals. This study is a systematic review to in order assess the prevalence of toxocariasis in North America and whether selected groups are at risk for infection. The prevalence was highest among Mexican children with asthma, but also African Americans living in poverty exhibited high rates of infection. Among the major risk factors for infection were African-American ethnicity, male sex, pet ownership, and environmental contamination by animal species. Additional high risk groups in Mexico included waste pickers, asthmatic children, and inpatient psychiatry patients. Further research is needed to determine the true current burden of toxocariasis in North America; however, the prevalence estimates gathered in this review suggest that the burden of disease is significant. Thus, toxocariasis may be emerging as an important health disparity in North America

## Introduction

Toxocariasis is an important neglected tropical disease caused by the roundworms *Toxocara canis* and *Toxocara cati*, which are transmitted when eggs in canine or feline feces, respectively, are ingested by humans [1]. Larvae penetrate the walls of the intestine then travel through the circulatory system to various organs throughout the body. *Toxocara spp.* do not multiply within the human host, but exist in host tissues in a state of arrested development [2], [3]. When larvae die, the inflammatory reaction produced by the body causes the symptoms of toxocariasis [1]. Toxocariasis can manifest in three ways; visceral larva migrans results from larvae migrating to major organs including the brain, lungs, and liver, causing a variety of signs and symptoms (usually in young children) including eosinophilia, abdominal pain, headache, cognitive and behavioral disturbances, pneumonitis, hepatitis with hepatomegaly, while ocular toxocariasis more commonly occurs in older children and results from larvae migrating to the eye and can cause irreversible vision loss due to scarring of the retina or retinal detachment. Covert toxocariasis, the most difficult form to diagnose, is believed to be caused by chronic exposure and can manifest as eosinophilia with cognitive disturbances, or nonspecific symptoms that resemble asthma, i.e., coughing, and wheezing [1], [2], [4], [5]. Both visceral larva migrans and covert toxocariasis, and often ocular larva migrans, are associated with enzyme immunoassay antibody titers that can remain elevated for months or years [6].

Toxocariasis causes significant morbidity and loss of productivity and poses an important, yet largely unaddressed public health problem in areas of high prevalence. Most notably, infection with *Toxocara spp.* has been associated with reduced cognitive function in children; Walsh and colleagues found that infected children scored significantly lower on math, reading, digit span, and block design tests than uninfected children [7]. Infected children were significantly more likely to be in a lower socioeconomic class than uninfected children, though the difference in performance persisted when socioeconomic class was controlled for, suggesting that toxocariasis, like many other neglected tropical diseases, may play a role in perpetuating poverty and contribute to health disparities in endemic areas [7]. It has been suggested that toxocariasis may even partially account for the achievement gap noted among socioeconomically disadvantaged students [8]. Medically, toxocariasis has also been proposed to be a potential cause of asthma, has been associated with seizures, and is an important cause of blindness [4], [9].

The current burden of disease due to toxocaraisis in North America is largely unknown. We conducted this review in order to determine the both the prevalence of and the amount of available data measuring the burden of toxocariasis in North America, and to identify needed areas of future research.

## Methods

### Search strategy and selection criteria

Searches were completed in February 2014 using PubMed and were restricted to English articles, articles published in the last 10 years, and articles pertaining to humans. Search terms included “toxocara”, “toxocariasis”, “visceral larva migrans”, and “ocular larva migrans” and the country name: “United States”, “Mexico”, and “Canada”. Searches were also performed using the country names and disease variations as MeSH terms. Non-English articles, dead links, and duplicate results were excluded from abstract review. Abstracts were reviewed by two reviewers and were excluded from full-text review if they were definitively about a different disease or about the relevant disease in an animal population.

### Assessment and data extraction

Documents in the full-text review were classified as containing (1) original prevalence data/estimates for toxocariasis, (2) original incidence or outbreak data, (3) reports of individual cases of toxocariasis, or (4) no original prevalence, incidence, outbreak, or case report information. Citations for unoriginal prevalence, incidence, or case report data were followed up and included in the review if they were English articles published within the past 10 years. Each paper was reviewed independently by two reviewers for figures, tables, or text containing original prevalence, incidence, and case data as well as descriptions of persons or populations affected and reported risk factors for disease. Articles using the same source data but reporting prevalence estimates for different age groups were considered to contain original estimates. Discrepancies between reviewers to resolved by discussion and consensus. This review is compliant with the PRISMA checklist for systematic reviews. Full bibliography available on request.

## Results

**Figure 1:**
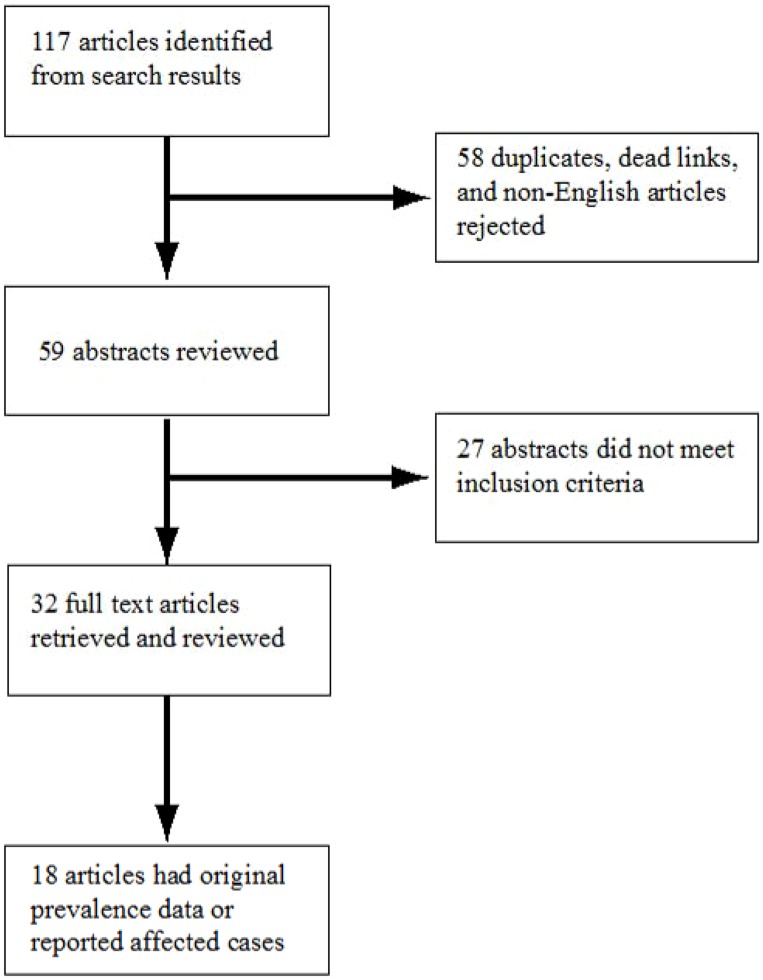
Systematic review process.

We found 18 articles with original prevalence, incidence, or case data for toxocariasis (Figure 1). Of these articles, 5 reported data in Canada, 8 reported data in the United States, and 5 reported data in Mexico. One article reported only prevalence data, 0 articles reported incidence or outbreak data, 5 articles reported only affected cases, and 12 articles reported both prevalence data and number of affected cases. Table 1 summarizes the prevalence and reported cases data for toxocariasis in North America. 63% (5/8) of articles with seroprevalence estimates for the United States tested blood samples collected during the 1988–1994 National Health and Nutrition Examination Survey (NHANES).

**Table 1:** Summary of *Toxocara spp.* seroprevalence estimates in published literature.

Country	Article	Year	Location	Seroprevalence (IgG)	N	Demographics
*Canada*						
	Campagna 2011 [26]	2007	Eastmain Village, James Bay, Northern Quebec	5.4%	6	Indiginous Cree community, volunteers ages 15 and older, 37% male
			Wemindji Village, James Bay, Northern Quebec	1.4%	2	Indiginous Cree community, volunteers ages 15 and older, 46% male
	Levesque 2007 [17]	2005	Mistissini Village, Lake Mistissini, Quebec	4.2%	2	Indiginous Cree community, active hunter/trappers and their spouses, 44% male
	Ota 2009 [27]	2008	Alberta	-	1	14 month old boy with history of retinoblastoma
	Sampasa-Kayinga 2012 [11]	2008	Chisasibi Village, James Bay, Northern Quebec	0.6% (95% CI 0.02–3.4%)	1	Indiginous Cree community, volunteers ages 18 and older, 41% male
			Waskaganish Village, James Bay, Northern Quebec	10% (95% CI 4.9–17.5%)	10	Indiginous Cree community, volunteers ages 18 and older, 42% male
	Schurer 2013 [12]	2011	Saskatchewan	13.4%	27	Indiginous Dene community, volunteers ages 4 and older, 62% male
*United States*						
	Congdon 2011 [16]	1988–1994	Countrywide	14.6% males, 12.6% females	2862	NHANES data, adults and children ages 6 and over
	Jones 2008 [3]	1988–1994	Countrywide	8.6%	63	NHANES data, children ages 1–5
				15.1%	240	NHANES data, children ages 6–11
	MMWR 2011 [15]	Sept 2009–Sept 2010	23 states, DC, and Puerto Rico *	-	68	Newly diagnosed ocular toxocariasis cases reported by ophthalmologists, median age 8.5 years (range 1–60 years), 56% male
	Stewart 2005 [21]	1977–1996	San Francisco, California	-	22	Ocular toxocariasis patients seen for uveitits, median age 14 years (range 1–37 years), 45.5% male
	Walsh 2011 [19]	1988–1994	Countrywide	14.2% (95% CI 12.7–15.9%)	1898	NHANES data, adults age 17–64
	Walsh 2012 [7]	1988–1994	Countrywide	-	688	NHANES data, children ages 6–16
	Won 2008 [10]	1988–1994	Countrywide	13.9% (95% CI 12.5–15.3%)	2835	NHANES data, adults and children over 6 years, 48.4% male
	Woodhall 2012 [20]	Sept 2009–Sept 2010	33 states, DC, and Puerto Rico**	-	159	Ocular toxocariasis patients reported by ophthalmologists, median age 11.5 years (range 1–66 years), 54% male
*Mexico*						
	Alvarado-Esquivel 2013 [13]	-	Durango City	13%	12	Waste pickers, mean age 36 years (range 14–76 years), 38% male
	Alvarado-Esquivel 2013 [14]	-	Durango City	4.7%	6	Inpatient psychiatric patients, mean age 43.6 (range 16–83), 69% e s
	Jimenez-Balderas 2012 [28]	Mexico City	16.6%	4	Ankylosing spondylitis patients, mean age 44 years, 38% male	
	Munoz-Guzman 2010 [9]	-	Mexico City	30.80%	-	Asthmatic children from the Traumatology Service of Federico Gomez Children's Hospital, mean age 6.9 years (range 4–12 years), 65% male
	Romero Nunez 2013 [18]	Aug 2010–Sept 2010	Municipality of Ecatepec of Morelos	22.2%	26	Volunteers age 2–16 years, 48% male

*Alabama (5), Arkansas (2), California (6), Connecticut (2), District of Columbia (3), Florida (8), Georgia (9), Illinois (3), Indiana (2), Iowa (1), Louisiana (1), Maryland (2), Nevada (2), New York (3), North Carolina (1), Ohio (1), Oklahoma (1), Oregon (1), Pennsylvania (1), Puerto Rico (1), South Carolina (3), Tennessee (1), Texas (16), Virginia (2), West Virginia (1).

**Alabama (6), Arkansas (2), California (7), Colorado (3), Connecticut (3), District of Columbia (8), Florida (10), Georgia (9), Hawaii (1), Idaho (2), Illinois (13), Indiana (6), Iowa (2), Louisiana (2), Maryland (4), Massachusetts (2), Michigan (2), Minnesota (2), Missouri (1), Nevada (3), New York (14), North Carolina (2), Ohio (10), Oklahoma (5), Oregon (1), Pennsylvania (6), Puerto Rico (1), Rhode Island (1), South Carolina (3), Tennessee (3), Texas (13), Utah (1), Virginia (3), Washington (7), West Virginia (1).

Data for the United States was the most robust, as many articles analyzed blood samples collected for NHANES, which collects data from a population representative of that of the United States. However, blood samples for the most recent NHANES were collected between 1988 and 1994 and thus prevalence data may not be representative of the current burden of disease 20 years later. Prevalence estimates for the United States ranged from 8.6% for ages 1–5 to 15.1% for ages 6–11, with an overall prevalence rate of 13.9% reported for all adults and children ages 6 and older [3], [10]. Data for Canada focused mainly on indigenous communities living in rural areas. Prevalence estimates ranged from 0.6% to 13.4%, indicating that for some communities toxocara infection causes a significant burden; however, the overall burden of disease in Canada as a whole is still unknown [11], [12]. Reported seroprevalence estimates in Mexico tended to be higher than those reported for the United States and Canada and the data available focused on comparing prevalence rates in high-risk groups, such as psychiatry patients, waste pickers, and asthmatic children to controls (Table 2) [9], [13], [14]. Seroprevalence rates in these high-risk groups ranged from 4.7% in inpatient psychiatric patients to 30.8% in children with asthma [9], [14].

**Table 2:** Case control studies of *Toxocara spp.* seroprevalence.

Article	Case Group	Case Group Seroprevalence (N)	Control Group	Control Group Seroprevalnce (N)	P value
Alvarado-Esquivel 2013 [13]	90 waste pickers aged 14–76 years, 38% male	13% (12)	90 non-waste pickers of various occupations matched by age and gender	1% (1)	<0.01
Alvarado-Esquivel 2013 [14]	128 psychiatric inpatients aged 16–83 years, 69% male	4.7% (6)	276 volunteers aged 16–91 years, 40% male	1.1% (3)	0.03
Jimenez-Balderas 2012 [28]	24 ankylosing spondylitis patients mean age 44±14 years, 38% male	16.6% (4)	77 healthy volunteers mean age 30±15 years, 40% male	2.6% (2)	0.027
Munoz-Guzman 2010 [9]	285 asthmatic children aged 4–12 years, 65% male	30.8% (88)	152 non-asthmatic children aged 4–12 years	19.7% (30)	<0.05

All case control studies identified were conducted in Mexico.

**Figure 2:**
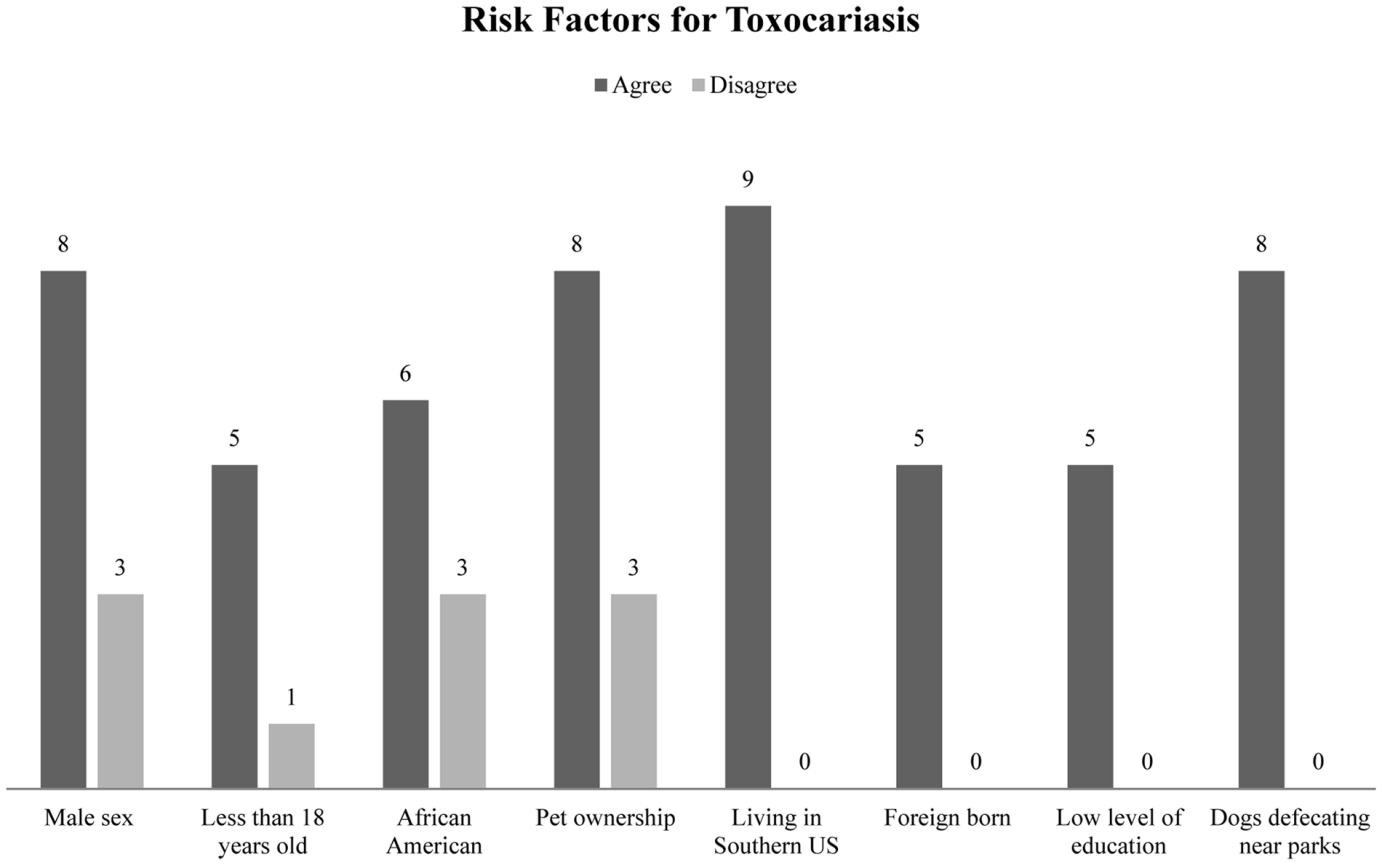
Articles citing risk factors for *Toxocara spp.* infection.

The most commonly cited risk factors for toxocariasis included male sex, canine or feline pet ownership, particularly if the animals are allowed to live primarily outdoors and eat other animals or otherwise unconventional pet food, African American race, age less than 18, low level of education, being foreign born, living in the southern United States, and playing at parks or in sandboxes where dogs and cats have defecated (Figure 2).

Twelve studies reported prevalence of *Toxocara spp.* antibodies by gender [2], [3], [7], [10], [11], [12], [15], [16], [17], [18], [19], [20]. Eight (73%) of these studies found a significantly greater prevalence in males compared to females, while 27% of studies found no significant differences between genders. No studies found females to have significantly higher prevalence of toxocara infection than males. Three studies from Canada reported prevalence by gender; two of these studies reported no significant difference in prevalence between genders and one study reported an increased prevalence in males [11], [12], [17]. Seven studies from the United States reported prevalence by gender; 7 of these studies reported an increased prevalence in males and one study reported no difference between genders [2], [3], [7], [10], [15], [16], [19], [20], [21]. One study from Mexico reported prevalence by gender; this study found a significantly higher prevalence in males than females [18].

Canine and feline ownership is a somewhat controversial yet commonly cited risk factor. Infected cats and dogs pass eggs in feces, which can contaminate their environment and be ingested by humans [1]. Puppies in particular are commonly infected in utero and must be dewormed after birth [1], [2]. Uninfected cats and dogs are at a high risk for becoming infected if they live outside or eat a diet of other animals or otherwise unconventional pet food [1]. These findings indicate that perhaps ownership itself is not an independent risk factor for toxocariasis, but that type of pet care can increase or decrease the risk of infection. Three studies from Canada indicated that pet ownership was not a risk factor, citing that dogs that are owned are more likely to be dewormed than feral dogs, which are more likely to eat an unconventional diet and live exclusively outside [11], [12], [17]. Additionally, environmental contamination by infected animals is an important risk factor; contamination rates of up to 40% have been found in urban playgrounds and children who play in public areas that are not kept clear of animal feces are at a higher risk for *Toxocara* infection [2], [20], [22].

All eight studies included in this review from the United States provided prevalence estimates by race. Five of these studies cited African American race as a risk factor [3], [7], [10], [16], [19]. Jones and colleagues found the odds ratio of Non-Hispanic Blacks being infected compared to Non-Hispanic Whites to be 1.7 (95% CI 1.4–2.0), while the odds ratio for Mexican-Americans compared to white was found to be 0.6 (95% CI 0.4–0.6) [3]. The national seroprevalence for ages 6 and over was estimated to be 22.8% in African Americans, 12.6% in Mexican-Americans, and 10.6% in Whites using the 1988–1994 NHANES data [10]. This disparity in prevalence between races has interesting public health implications. Hotez et al. estimated that as many as 2.8 million African-Americans living in poverty in the United States were infected with *Toxocara spp.* making toxocariasis one of the most common infections in an underrepresented minority group [23]. The three studies that reported an increased prevalence of toxocariasis in Whites compared to African-Americans were studies that involved surveying clinicians treating patients with uveitis and asking them to report cases presented with ocular toxocariasis [15], [20], [21]. The discrepancy between these two sets of studies may point to an issue of access to healthcare in the African-American community.

## Discussion

The findings in this review indicate that the seroprevalence of human toxocariasis is high in North America, frequently exceeding 10 percent in the United States and Mexico and among some populations in Canada. Moreover, they suggest that toxocariasis may be emerging as a North American health disparity, with the highest seroprevalence found among African Americans and children in the southern U.S. and some indigenous communities in Canada, as well as populations with low education levels those living in areas where dog feces are found, indicative of environmental degradation.

The detection of antibodies against Toxocara antigens in sera is associated with past exposure to *T. canis* and *T. cati* eggs and the resulting parasite larval migrations. However, because Toxocara larvae can remain in a developmentally arrested state and live with minimal metabolic activity in mammalian tissues for years [24], seropositivity – enzyme immunoassay titers can remain positive for years [6] - may also be indicative of active infection. This finding can help explain the associations noted in case-control studies conducted in Mexico between Toxocara seropositivity and conditions such as asthma and ankylosing spondylitis, and psychiatric illness [7], [11], [12], [24], and US NHANES data linking toxocariasis to pulmonary disease and cognitive deficits [6], [17].

Limitations of this study include restrictions used in our search methodology, in particular only including articles published within the past 10 years. An additional limitation was that the studies conducted in the US mostly relied on the same NHANES data. Another was the lack of harmonization of study populations between the different North American countries. For example all of the Canadian studies were conducted in adults only.

Our overall purpose was to assess the available literature on the current burden of disease and to discern the amount of evidence available to inform current or future public health measures to control and prevent toxocariasis. Ultimately, more recent and widespread research is needed to determine the true current burden of toxocariasis in North America, but the prevalence estimates gathered during this review preliminarily indicate that the burden of disease is not insignificant. Other particularly salient points identified by this review that would benefit from further research efforts include differences in prevalence by race in the United States, especially with how these differences are confounded by or related to income and education levels, the relationship between toxocariasis and asthma in children and between toxocariasis and psychiatric diagnoses in both children and adults, and the cognitive effects of toxocara infection in childhood. Links between arthritis and toxocariasis also require further investigation.

Recently the US Centers for Disease Control and Prevention (CDC) announced an initiative to prioritize five neglected parasitic infections in the US, including toxocariasis [25]. Among the major priorities outlined by the CDC are efforts to better define risk factors, and research to elucidate the natural history of toxocariasis and its provocative associations with pulmonary and neurologic diseases [6]. There is also urgency to develop improved and more widely accessible diagnostic tests for detecting active infection, and studies to optimize current treatment regimens using anthelminthic drugs [6]. Thus, we are still in the nascent stages of understanding the full extent of human toxocariasis in the US and North America and how to best diagnose, manage and treat, and ultimately prevent this illness and emerging health disparity.

## Supporting Information

Text S1 **PRISMA checklist.**
(DOC)

Text S2 **PRISMA flowchart.**
(DOC)

Text S3 **Full reference list.**
(XLSX)
