# Supplementary material for: Toxocariasis in North America: A Systematic Review
Source: PLoS Negl Trop Dis. 2014 Aug 28;8(8):e3116. doi: 10.1371/journal.pntd.0003116 (PMC4148222; doi:10.1371/journal.pntd.0003116)
Supplement: Text S2 — PRISMA flowchart. (DOC) [file pntd.0003116.s002.doc]

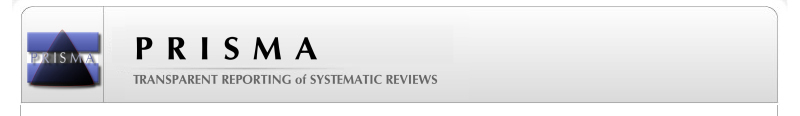
**PRISMA 2009 Flow Diagram**

**Screening**

**Included**

**Eligibility**

**Identification**

Articles identified through database searching
(n =119 )

Additional articles identified through other sources
(n = 5)

Articles after duplicates removed
(n = 61)

Abstracts screened
(n = 61)

Records excluded because non-English articles, unrelated, or dead links
(n =24 )

Full-text articles assessed for eligibility
(n = 37)

Full-text articles did not meet inclusion criteria
(n = 9)

Articles included in paper
(n = 28)

Studies had original prevalence date or reported affected cases
(n = 18)
